# Molecular insights into HER2/*ERBB2* amplification and carcinogenesis in gallbladder cancer associated with pancreaticobiliary maljunction

**DOI:** 10.1007/s00535-025-02303-2

**Published:** 2025-10-04

**Authors:** Ming Zhu, Daisuke Douchi, Keigo Murakami, Taito Itoh, Shusuke Migita, Naoki Rikiyama, Shuichiro Hayashi, Takashi Kokumai, Hideaki Sato, Shingo Yoshimachi, Akiko Kusaka, Mitsuhiro Shimura, Shun Nakayama, Shuichi Aoki, Masahiro Iseki, Takayuki Miura, Shimpei Maeda, Masaharu Ishida, Hideo Ohtsuka, Masamichi Mizuma, Kei Nakagawa, Atsushi Masamune, Toru Furukawa, Michiaki Unno

**Affiliations:** 1 Department of Surgery, Tohoku University Graduate School of Medicine, 1-1 Seiryo-Machi, Aoba-Ku, Sendai, Miyagi Japan; 2 Department of Investigative Pathology, Tohoku University Graduate School of Medicine, 1-1 Seiryo-Machi, Aoba-Ku, Sendai, Miyagi Japan; 3 Laboratory of Molecular Diagnostics and Therapeutics, Center for Intractable Diseases and ImmunoGenomics, National Institutes of Biomedical Innovation, Health and Nutrition, 7-6-8 Saito-Asagi, Ibaraki, Osaka Japan; 4 Division of Gastroenterological and Hepato-Biliary-Pancreatic Surgery, Department of Surgery, Tohoku Medical and Pharmaceutical University, 1-15-1 Fukumuro, Miyagino-Ku, Sendai, Miyagi Japan; 5 Division of Gastroenterology, Tohoku University Graduate School of Medicine, 1-1 Seiryo-Machi, Aoba-Ku, Sendai, Miyagi Japan

**Keywords:** Pancreaticobiliary maljunction, Gallbladder cancer, HER2, *ERBB2*

## Abstract

**Background:**

Pancreaticobiliary maljunction (PBM) contributes to epithelial hyperplasia and, ultimately, the development of gallbladder cancer (GBC). Despite its clinical significance, the molecular and cellular mechanisms driving carcinogenesis in GBC with PBM remain poorly elucidated. This study investigated the oncogenic mechanisms, biomarkers, and performance associated with Erb-b2 receptor tyrosine kinase 2 (*ERBB2)*-targeted therapies in GBC with PBM.

**Methods:**

Overall, 127 surgically treated patients were stratified as follows: Group A, normal gallbladder; Group B, PBM; Group C, GBC without PBM; and Group D, GBC with PBM. We performed whole-exome sequencing (WES) for Group D and human epidermal growth factor receptor 2 immunohistochemistry (HercepTest) for the entire cohort. Fluorescence in situ hybridization (FISH) was used to clarify human epidermal growth factor receptor 2 (HER2) expression in cases with equivocal HercepTest results.

**Results:**

*ERBB2* amplification was detected in 50% of Group D patients. The proportion of HER2 protein expression scores ≥ 2 + was highest in Group D compared with that in the other groups (50.0% vs. 0% in Groups A and B and 15.6% in Group C) (*P* = 0.006, chi-squared test). Finally, 37.5% and 13.3% of cases in Groups D and C, respectively, showed HER2 overexpression (*P* = 0.037, chi-squared test).

**Conclusions:**

This is the first evaluation of HER2/*ERBB2* expression in GBC with PBM based on WES, HercepTest, and FISH. The significant increase in *ERBB2* expression, driven by the synergistic interplay between GBC and PBM, underscores a critical molecular interaction that may inform the development of targeted therapeutic strategies.

**Supplementary Information:**

The online version contains supplementary material available at 10.1007/s00535-025-02303-2.

## Introduction

Gallbladder cancer (GBC) is an aggressive malignancy with a poor prognosis and limited treatment options [1, 2]. Early stage GBC frequently presents with no or nonspecific symptoms; thus, delayed or misdiagnosis is common, and some diagnoses are only made after cholecystectomy for symptomatic gallstones [3, 4]. Owing to its aggressive nature, GBC rapidly infiltrates adjacent structures, including regional lymph nodes and the liver, and metastasizes to the peritoneum, resulting in a poor prognosis [5, 6]. The 5-year survival rate remains relatively low due to the advanced stage at diagnosis and limited effective treatment options [7, 8].

Chronic gallbladder inflammation plays a pivotal role in GBC pathogenesis and is closely associated with pancreaticobiliary maljunctions (PBMs) [3, 5]. A PBM is an abnormal connection between the pancreatic and common bile ducts, forming a common channel outside the duodenal wall that results in unregulated reflux of pancreatic juice into the biliary system [9]. This reflux, known as regurgitation, occurs when the junction is beyond the control of the sphincter of Oddi [10]. The reflux of pancreatic enzymes and bile into the gallbladder induces chronic inflammation, causing repeated cycles of epithelial damage and repair [11, 12], eventually leading to molecular (genetic and epigenetic) changes that can initiate and promote carcinogenesis [3, 12]. The pathophysiological mechanisms underlying PBM development contribute to GBC risk. Most investigations into PBMs have been conducted in Asian populations, particularly in East Asia, where it may present in 1 in every 1000 individuals [13]. In contrast, reports from Western countries remain limited; although the precise prevalence is unclear, it has been estimated at approximately 1 in 100,000 individuals [14]. Some East Asian and Western populations have shown comparable prevalences [15]. In a cohort study of 300 patients with GBC in the USA, the prevalence of PBM was 8.0%, comparable to the 8.8% in Japan [15]. Despite the well-established role of PBM as a risk factor for biliary malignancies [10], further genetic and molecular analyses are needed to elucidate its oncogenic potential.

Genetic alterations can contribute to a poor prognosis, including mutations in Erb-b2 receptor tyrosine kinase 2 (*ERBB2*), Erb-b2 receptor tyrosine kinase 3 (*ERBB3*), tumor protein p53 (*TP53*) and E74-like ETS transcription factor 3 [16]. *ERBB2* is a proto-oncogene of the epidermal growth factor receptor (*EGFR*) family located on the long arm of chromosome 17 [17]. *ERBB2* generally refers to the gene, whereas human epidermal growth factor receptor 2 (HER2) denotes the protein product. Structural and functional alterations in *ERBB2* occur at different stages of carcinogenesis, including initiation, promotion, and progression [18, 19]. The most frequent alteration involves increased *ERBB2* copy number [20, 21]. However, no studies have specifically investigated *ERBB2* alterations in GBC with PBM.

In this study, we performed a genetic and molecular characterization of GBC presenting with PBM. We aimed to elucidate the underlying oncogenic mechanisms, potential biomarkers, and clinical performance of *ERBB2*-targeted therapeutic strategies.

## Methods

### Patients

This study included patients who underwent surgical procedures at the Department of Surgery, Tohoku University Hospital (Sendai, Japan), between 2010 and 2024. Clinicopathological data were retrospectively extracted from electronic medical records. The Institutional Review Board of Tohoku University in 2021 approved the research protocol (approval no. 2021–1–864) and waived the requirement for informed consent due to the retrospective nature of the study. An opt-out approach was implemented, and the study was conducted in accordance with the principles outlined in the Declaration of Helsinki. Tumor staging was performed using the 8th edition of the Union for International Cancer Control TNM classification of malignant tumors.

### PBM definition

PBM was defined according to the criteria established by the Japanese Study Group on Pancreaticobiliary Maljunction in 2013 [22]. PBM is characterized by (A) an anomalous union of the main pancreatic and common bile ducts occurring outside the sphincter of Oddi and beyond its regulatory control, as confirmed via direct cholangiography, or (B) an extramural anatomical configuration outside the duodenal wall. The diagnosis of PBM was confirmed based on either (1) direct cholangiography [intraoperative or endoscopic retrograde cholangiopancreatography (ERCP)] showing a common channel located outside the duodenal wall and beyond the control of the sphincter of Oddi, or (2) cross-sectional imaging, including magnetic resonance cholangiopancreatography, demonstrating an anomalous junction of the pancreatic and bile ducts. Experienced hepatobiliary radiologists and surgeons reviewed all imaging data, with the final diagnosis made through multidisciplinary consensus.

### Whole-exome sequencing (WES)

The molecular basis of GBC with PBM was investigated using formalin-fixed paraffin-embedded (FFPE) tissue sections from GBC specimens. Serial 10-μm-thick sections were prepared from FFPE blocks using a microtome exclusively for DNA extraction. Genomic DNA was isolated using the GeneJET FFPE DNA Purification Kit (Thermo Fisher Scientific, Waltham, MA, USA), following the manufacturer’s instructions. WES was performed by Macrogen Japan Corp. (Tokyo, Japan). Overall, 200 ng of gDNA per sample was amplified, and libraries were constructed using a SureSelect Human All Exon V6 kit (Agilent Technologies, Santa Clara, CA, USA). DNA sequencing was performed on an Illumina NovaSeq 6000 (Illumina, San Diego, CA, USA) with a 150-bp paired-end read length. Analysis was initiated from FASTQ data using the nf-core/sarek pipeline (version 3.3.0) [23]. Adapter sequences and low-quality reads in the FASTQ data were filtered with fastp (version 0.23.2) [24] and aligned to the human reference genome (hs37d5) using BWA–MEM (version 0.7.17) [25]. The aligned reads were processed using GATK MarkDuplicates (version 4.3.0.0), GATK BaseRecalibrator, and GATK ApplyBQSR [26]. Somatic variant calling was performed using GATK Mutect2. Somatic mutations were determined by comparing independent genotyping results between tumor and normal lymph node samples. PureCN (version 2.8.1) [27] was used for copy number variation (CNV) detection, and copy number alterations were categorized as follows: 0, deep deletion; 1, shallow deletion; 3–4, shallow gain; and ≥ 5, amplification. Mutational and copy number signatures were extracted using SigProfilerExtractor [28]. Additional analyses, including somatic mutation enrichment and focal copy number Genomic Identification of Significant Targets in Cancer (GISTIC) profiling, were conducted using the maftools package [29, 30].

### HER2 immunohistochemistry (HercepTest)

**Fig. 1:**
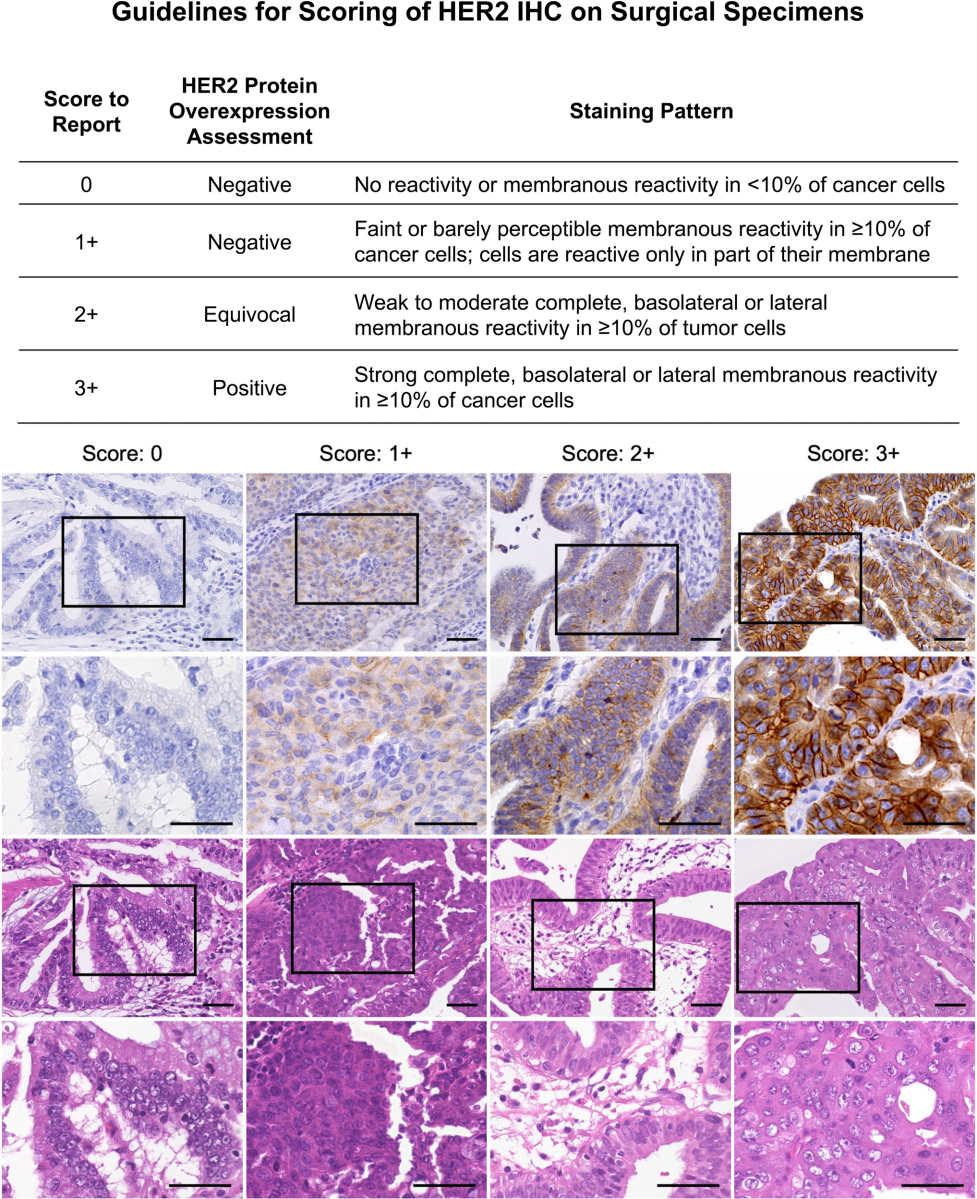
Guidelines for HercepTest scoring of surgical specimens. Representative scoring images from four GBC specimens are shown. For each scoring category, four paired images are displayed in a single column, illustrating the same field with corresponding HercepTest and H&E staining. Boxes indicate enlarged regions. Scale bars = 50 µm. *GBC* Gallbladder cancer, *H&E* Hematoxylin and eosin, *HER2* Human epidermal growth factor receptor 2

Hematoxylin and eosin (H&E) staining and HercepTest were performed on all FFPE tissue blocks to assess the association between HER2 expression and GBC. Sections (4-μm thick) were cut from FFPE blocks using a microtome. HercepTest assays were conducted on whole-tissue sections using HercepTest™ (Agilent, Dako, Carpinteria, CA, US) on a Dako Autostainer (Dako), following the manufacturer’s protocol. The HER2 scoring system followed the HercepTest criteria for gastric cancer [21, 31, 32]. Given the intratumoral heterogeneity in GBC, which is similar to that in gastric cancer, staining patterns other than complete membrane staining—incomplete, basal, or lateral membrane staining—were also considered positive [31–33]. HER2 expression was categorized as follows, based on examination at 40 × magnification using a Keyence microscope: 0 (negative, no reactivity or membranous reactivity in < 10% of cells), 1 + (negative, faint/barely perceptible membranous reactivity in at least 10% of cells or reactivity in only part of the cell membrane), 2 + (equivocal, weak-to-moderate complete or basolateral membranous reactivity in at least 10% of tumor cells), and 3 + (positive, strong complete or basolateral membranous reactivity in at least 10% of tumor cells) (Fig. 1). Samples with an immunohistochemistry (IHC) score of 2 + underwent further evaluation using fluorescence in situ hybridization (FISH) [34]. One trained pathologist performed IHC analysis. A second pathologist re-analyzed ambiguous cases.

### FISH

Tumors (IHC 2 +) were analyzed for gene amplification using FISH with a PathVysion HER2 DNA Probe Kit (Abbott, Chicago, IL, USA). Slides were hybridized with HER2/neu probes and chromosome enumeration probe 17 (CEP17, serving as a centromere marker), following the manufacturer’s instructions. Counterstaining was performed using 4′,6-diamidino-2-phenylindole. The slides were visualized using a fluorescence microscope. The HER2-to-CEP17 ratio was calculated following the manufacturer’s guidelines and was interpreted using the American Society of Clinical Oncology/College of American Pathologists guidelines [35]. Tumors with an HER2:CEP17 ratio of ≥ 2:1 were classified as HER2-positive.

### RNA sequencing (RNA-seq) and data analysis

Total RNA was extracted from the mucosal epithelium of freshly resected gallbladder specimens using a NucleoSpin RNA Kit (Takara Biotechnology, Tokyo, Japan), following the manufacturer’s protocol. Fresh tissue samples were immediately processed after surgical resection to preserve RNA integrity. RNA concentrations were measured using a NanoDrop One spectrophotometer (Thermo Fisher Scientific). RNA-seq was performed on an Illumina NovaSeq 6000 platform (Macrogen, Seoul, Republic of Korea). Normalized gene expression data were analyzed using gene set enrichment analysis (GSEA) with the Broad Institute’s GSEA tool (http://software.broadinstitute.org/gsea/index.jsp) and Molecular Signatures Database (MSigDB, version 2024.1) to identify enriched gene sets and pathways. Differentially expressed genes (DEGs) were identified based on gene expression levels using DESeq2 and PoissonDis. Functional pathway enrichment analysis was performed using the phyper function in R software (The R Foundation, Vienna, Austria), with false discovery rates below 0.05 indicating significant enrichment. Functional enrichment analysis of all DEGs was performed using the Gene Ontology database (enriched terms were identified at a threshold of *P* < 0.05). The biological processes were further categorized to elucidate the underlying molecular mechanisms.

### Statistical analysis

Statistical analyses were performed using GraphPad Prism (version 10; GraphPad Software, San Diego, CA, USA). Data were analyzed using two-tailed chi-squared tests, with statistical significance set at *P* < 0.05. The Kruskal–Wallis *H* test was employed to compare continuous or ordinal variables among independent groups when normality or homogeneity of variance assumptions were not satisfied. A *P* value of < 0.05 was considered statistically significant.

## Results

### Patient cohort details

This study included 127 patients who underwent surgical treatment [mean age: 62 years, range: 16–89 years; 64 (50.4%) male, 63 (49.6%) female]. The study population was categorized into the following four groups: Group A (patients with normal gallbladders, without PBM, but with cholelithiasis and/or acute cholecystitis, *n* = 33), Group B (patients with PBM without biliary tract cancer, *n* = 33), Group C (patients with GBC without PBM, *n* = 45), and Group D (patients with GBC with PBM, *n* = 16) (Table 1).

**Table 1:** Clinicopathological features of GBC with or without PBM patient

	No. of patients (%)/median [range]					
	Total (*n* = 127)	A Normal (*n* = 33)	B PBM (*n* = 33)	C GBC without PBM (*n* = 45)	D GBC with PBM (*n* = 16)	*p value*^a^
Age (years)	62 [16–89]	59 [30–82]	41 [16–75]	71 [43–89]	62.5 [33–76]	0.001^b^
Sex						
Male	64 (50.4)	21 (63.6)	6 (18.2)	34 (75.6)	4 (25.0)	0.004^c^
Female	63 (49.6)	12 (36.4)	27 (81.8)	11 (24.4)	12 (75.0)	
Definitive diagnostic method of PBM						
ERCP	96 (75.6)	12 (36.4)	32 (97.0)	37 (82.2)	15 (93.8)	0.480^c^
MRCP only	31 (24.4)	21 (63.6)	1 (3.0)	8 (17.8)	1 (6.2)	
Bile duct dilation						
With (dilated type)	29 (59.2)	NA	25 (75.8)	NA	4 (29.4)	
Without (nondilated type)	20 (40.8)	NA	8 (24.2)	NA	12 (70.6)	
Operation procedure						
Simple cholecystectomy	46 (36.2)	33 (100)	4 (12.1)	9 (20.0)	0	< 0.001^c^
Extrahepatic bile duct resection	26 (20.5)	0	25 (75.8)	0	1 (6.3)	
Radical cholecystectomy	26 (20.5)	0	2 (6.1)	18 (40.0)	6 (37.4)	
EC + WRS	8 (6.3)	0	0	4 (8.9)	4 (25.0)	
RH + EBR	9 (7.1)	0	1 (3.0)	6 (13.3)	2 (12.5)	
Pancreatoduodenectomy	2 (1.6)	0	1 (3.0)	1 (2.2)	0	
Hepatopancreatoduodenectomy	4 (3.1)	0	0	3 (6.7)	1 (6.3)	
Others	6 (4.7)	0	0	4 (8.9)	2 (12.5)	
Cholelithiasis						
Present	49 (38.6)	30 (90.9)	5 (15.2)	14 (31.1)	0	0.010^c^
Absent	78 (61.4)	3 (9.1)	28 (84.8)	31 (68.9)	16 (100)	
Sample analyzed						
Primary	57 (93.4)	NA	NA	42 (93.3)	15 (93.8)	< 0.001^c^
Metastasis	4 (6.6)	NA	NA	3 (6.7)	1 (6.2)	
Histopathological subtype						
Adenocarcinoma	52 (85.3)	NA	NA	38 (84.5)	14 (87.4)	< 0.001^c^
Adenosquamous carcinoma	5 (8.2)	NA	NA	5 (11.1)	0	
ICPN with invasive carcinoma	3 (4.9)	NA	NA	2 (4.4)	1 (6.3)	
Adenoneuroendocrine carcinoma	1 (1.6)	NA	NA	0	1 (6.3)	
UICC TNM stage						
0–II	24 (39.3)	NA	NA	20 (44.4)	4 (25.0)	0.323^c^
III	19 (31.2)	NA	NA	12 (26.7)	7 (43.8)	
IV	18 (29.5)	NA	NA	13 (28.8)	5 (31.2)	

*PBM* Pancreaticobiliary maljunction, *GBC* Gallbladder Cancer, *ERCP* Endoscopic retrograde cholangiopancreatography, *MRCP* Magnetic resonance cholangiopancreatography, *EC* + *WRS* Extended cholecystectomy with wedge resection of segments IVb and V, *RH* + *EBR* Right hepatectomy with extrahepatic bile duct resection, *ICPN* Intracholecystic papillary neoplasm, *UICC* Union for international cancer control, *NA* Not applicable

^a^*P* values are reported for variables measured in the GBC without PBM group and the GBC with PBM group

^b^Kruskal–Wallis *H* test

^c^chi-squared test

Women predominated in Groups B and D, whereas men were more common in Groups A and C. Patients with PBM tended to be younger (median age: 41 years), whereas those with GBC without PBM were generally older (median age: 71 years). Most patients with PBM were diagnosed through ERCP. Bile duct dilation was noted in most patients of Group B, whereas the nondilated type predominated in Group D. In Groups A and B, simple cholecystectomy and extrahepatic bile duct resection were the predominant surgical approaches, while radical cholecystectomy was more frequently employed in Groups C and D. Among histopathological subtypes, adenocarcinomas were the most frequently observed across groups. Other subtypes, including adenosquamous carcinoma and intracholecystic papillary neoplasm with invasive carcinoma, were less common, with adenoneuroendocrine carcinoma observed only in Group D. Most GBC cases were primary and presented with TNM stage III or higher. Additional data are provided in Table 1.

### WES results for GBC with PBM cases

**Fig. 2:**
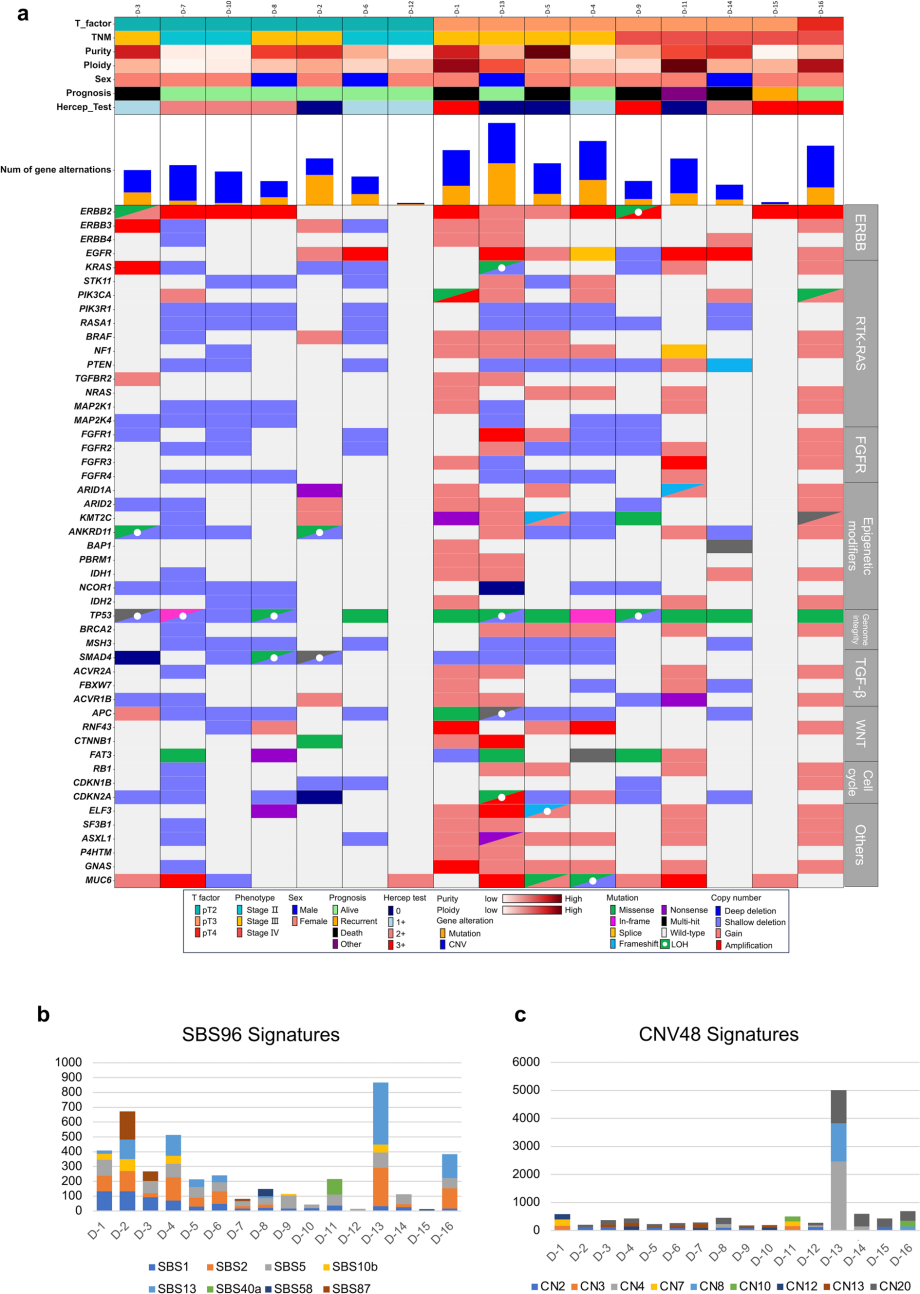
Integrated genomic and clinical landscape with mutational and CNV signatures in GBC with PBM. **a** Oncoprint of 16 resected GBC with PBM samples displaying clinical annotations and somatic alterations. The top annotation tracks show: T factor (pT2, pT3, and pT4), overall TNM stage (II, III, and IV), tumor purity and ploidy (color gradients from low to high), patient sex (male/female), clinical outcome (alive, recurrent, and deceased), and HER2 immunohistochemistry score (0–3 +). The bar plot immediately below indicates the total number of gene alterations per sample, subcategorized into somatic mutations (orange) and copy-number variants (blue). The main matrix summarizes gene-level events across a panel of cancer-related genes grouped based on pathway—ERBB signaling, RTK/RAS, FGFR signaling, epigenetic modifiers, genome integrity, TGF-β, WNT, and cell cycle, among others. Mutation types (missense, nonsense, in-frame indel, splice-site, frameshift, multi-hit, and loss of heterozygosity) and copy-number changes (deep deletion, shallow deletion, gain, and amplification) are color-coded according to the inset legend; wild-type genes are shown in light gray. **b** Stacked bar chart of single-base substitution (SBS96) signature contributions across the same samples, with signatures SBS1, SBS2, SBS5, SBS10b, SBS13, SBS40a, SBS58, and SBS87. **c** Stacked bar chart of copy-number (CNV48) signature contributions across the samples, with signatures CN2, CN3, CN4, CN7, CN8, CN10, CN12, CN13, and CN20. Mutational and copy-number signatures in panels (**b**) and (**c**) were extracted using SigProfilerExtractor. *GBC* Gallbladder cancer, *PBM* Pancreaticobiliary maljunction, *HER2* Human epidermal growth factor receptor 2, *ERBB* Erb-b2 receptor tyrosine kinase

WES was performed on 16 samples from Group D using matched tumor-normal pairs. The oncoplot of driver genes and somatic mutations associated with biliary tract cancer is shown in Fig. 2a. Copy number alterations were observed predominantly in *ERBB2* (68.8%); mucin 6, oligomeric mucus/gel-forming (50%); *EGFR* (43.8%); additional sex combs-like 1 (37.5%); guanine nucleotide-binding protein, alpha stimulating (37.5%); phosphatidylinositol-4,5-bisphosphate 3-kinase catalytic subunit alpha (37.5%); and *ERBB3* (31.3%), among others. Other genes, including *TP53*, exhibited major variations with a somatic mutation rate of 68.8%, whereas the somatic mutation rate in Kirsten ras was 6.3%. This high prevalence of *TP53* mutations is consistent with previous findings in GBC with PBM [36]. The predominant alterations in *ERBB2* were amplification (*n* = 8, 50.0%) and shallow gain (*n* = 3, 18.8%). The amplification rate for *EGFR* was 25% and reached 81.3% for the *ERBB* family, including *EGFR*, *ERBB1*, *ERBB2*, and *ERBB3*, with an overall frequency of copy number alteration as high as 93.8% in *ERBB* family. Analysis of SBS96 signatures showed that SBS1, SBS2, SBS5, and SBS13, among others, were frequently observed across Group D samples. Sample D-13 exhibited a notably high contribution from SBS2, SBS5, and SBS13. CNV48 signature analysis revealed generally low CNV activity in most samples, except for D-13, which showed markedly elevated CN4, CN8, and CN20 signatures (Fig. 2b, c).

**Fig. 3:**
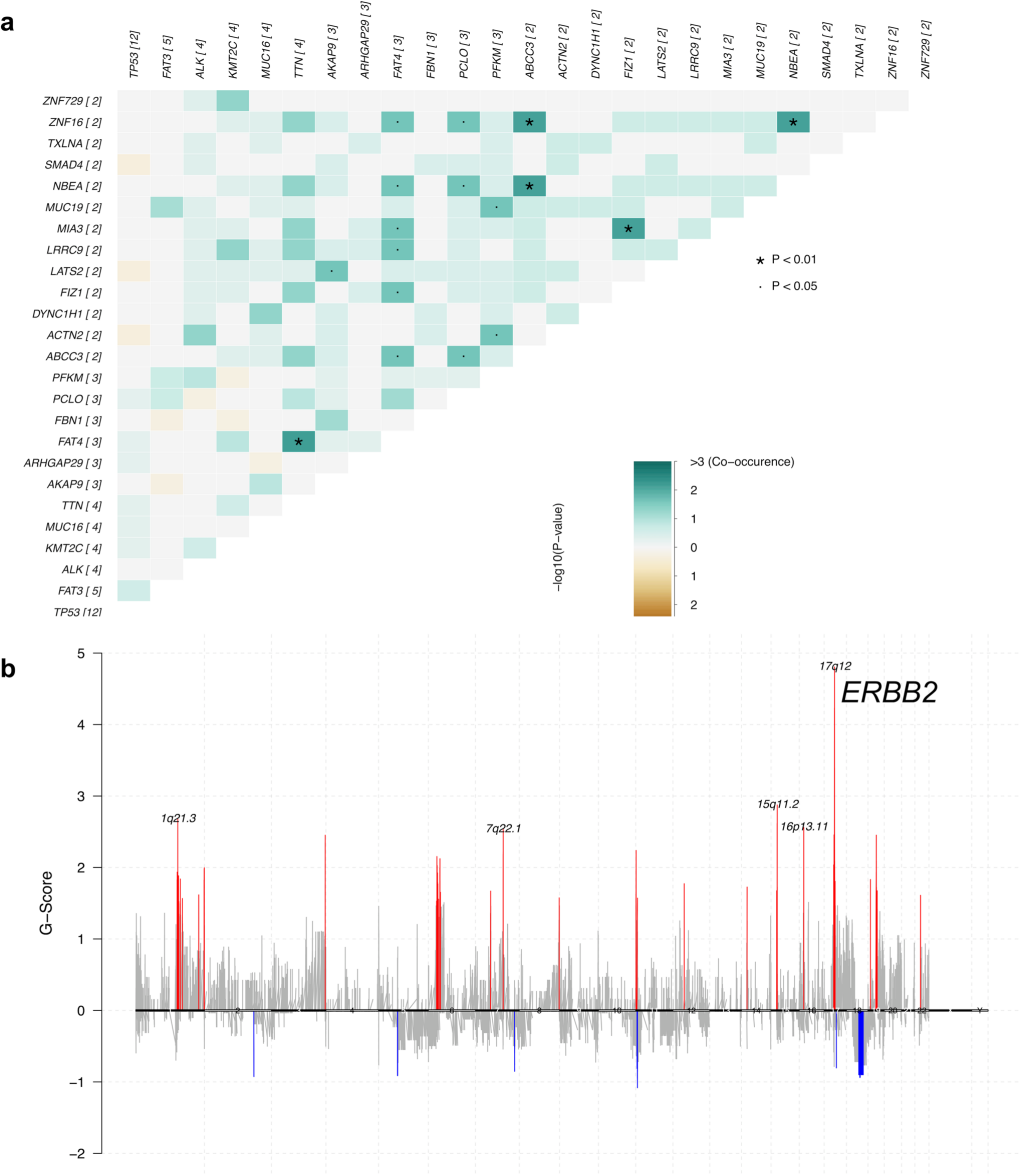
Pairwise mutation interactions and genome-wide copy-number GISTIC analysis. **a** Somatic interaction heatmap of recurrently mutated genes (genes mutated in ≥ 2 samples; mutation count per gene shown in brackets). Genes are ordered by decreasing mutation frequency. For each gene pair, the color intensity reflects the number of co-occurrence events (darkest green: > 3 co-occurrences) and the statistical significance of the association (–log₁₀P from Fisher’s exact test), with green and brown shades indicating co-occurrence and mutual exclusivity, respectively. Asterisks (*) denote *P* < 0.01 and dots (·) represent *P* < 0.05. **b** Genome-wide GISTIC2.0 G-score plot of somatic copy-number alterations. The *x*-axis spans chromosomes 1–22, and the *y*-axis shows the G-score. Positive peaks in red represent regions of recurrent amplification; negative troughs in blue represent recurrent deletions. Prominent focal peaks are annotated by cytogenetic band. *GISTIC* Genomic identification of significant targets in cancer

As shown in Fig. 3a, a somatic interaction heatmap was generated for recurrently mutated genes, ordered by decreasing mutation frequency. Notably, several gene pairs exhibited strong co-mutation patterns with high statistical significance. For example, *ZNF16* and *ABCC3*, *NBEA* and *ABCC3*, *MIA3* and *FIZ1*, *FAT4* and *TTN*, and *NBEA* and *ZNF16,* demonstrated significant co-occurrence (*P* < 0.01). These findings suggest potential cooperative oncogenic interactions or shared pathway involvement among these genes. Copy number analysis using GISTIC2.0 identified several significantly recurrent somatic copy-number alterations across the cohort (Fig. 3b). 17q12 exhibited the highest G-score, corresponding to the *ERBB2* locus, suggesting it as a potential focal amplification target. This region represents putative driver events involved in tumorigenesis.

Comparison of somatic mutation enrichment and focal copy-number alterations was performed between the GBC with PBM cases in Group D (*n* = 16) and a reference GBC cohort (*n* = 237) from MSK (Memorial Sloan Kettering Cancer Center) [37]. *FAT3* mutations were significantly enriched in the GBC with PBM cohort, with a mutation frequency of 31.3% (5/16), compared to 0% (0/237) in the MSK cohort (*P* < 0.001), yielding an infinite odds ratio. Although *KMT2C* and *ANKRD11* mutations were more prevalent in the GBC with PBM cohort than in the reference cohort, with odds ratios of 3.588 and 5.427, respectively, the differences did not reach statistical significance (Figure S1a).

In the GBC with PBM cohort, distinct amplification peaks were observed at 1q21.3, 7q22.1, 15q11.2, 16p13.11, and especially 17q12 (harboring *ERBB2*). Notably, regions such as 17q12 displayed amplification signals in both cohorts, indicating possible shared oncogenic drivers, although the amplitude and frequency differed. The *ERBB2* locus (17q12) exhibited a higher frequency of focal amplification in the GBC with PBM cohort than in the MSK GBC cohort (Figure S1b). Furthermore, analysis of focal deletion patterns also revealed both overlapping and distinct regions between the two cohorts, highlighting notable differences in the genomic profiles of the GBC with PBM and GBC cohorts.

### HercepTest results

**Fig. 4:**
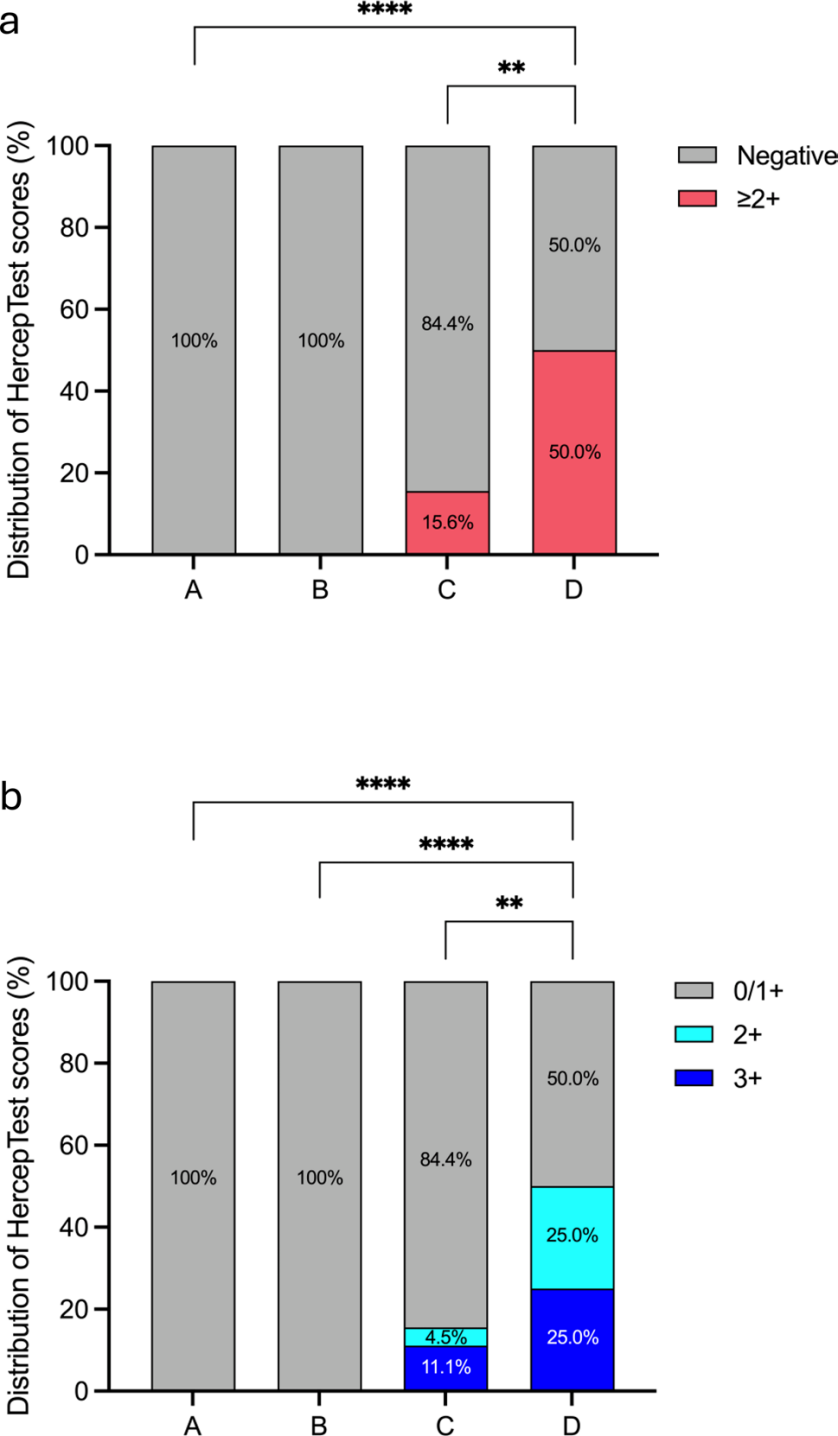

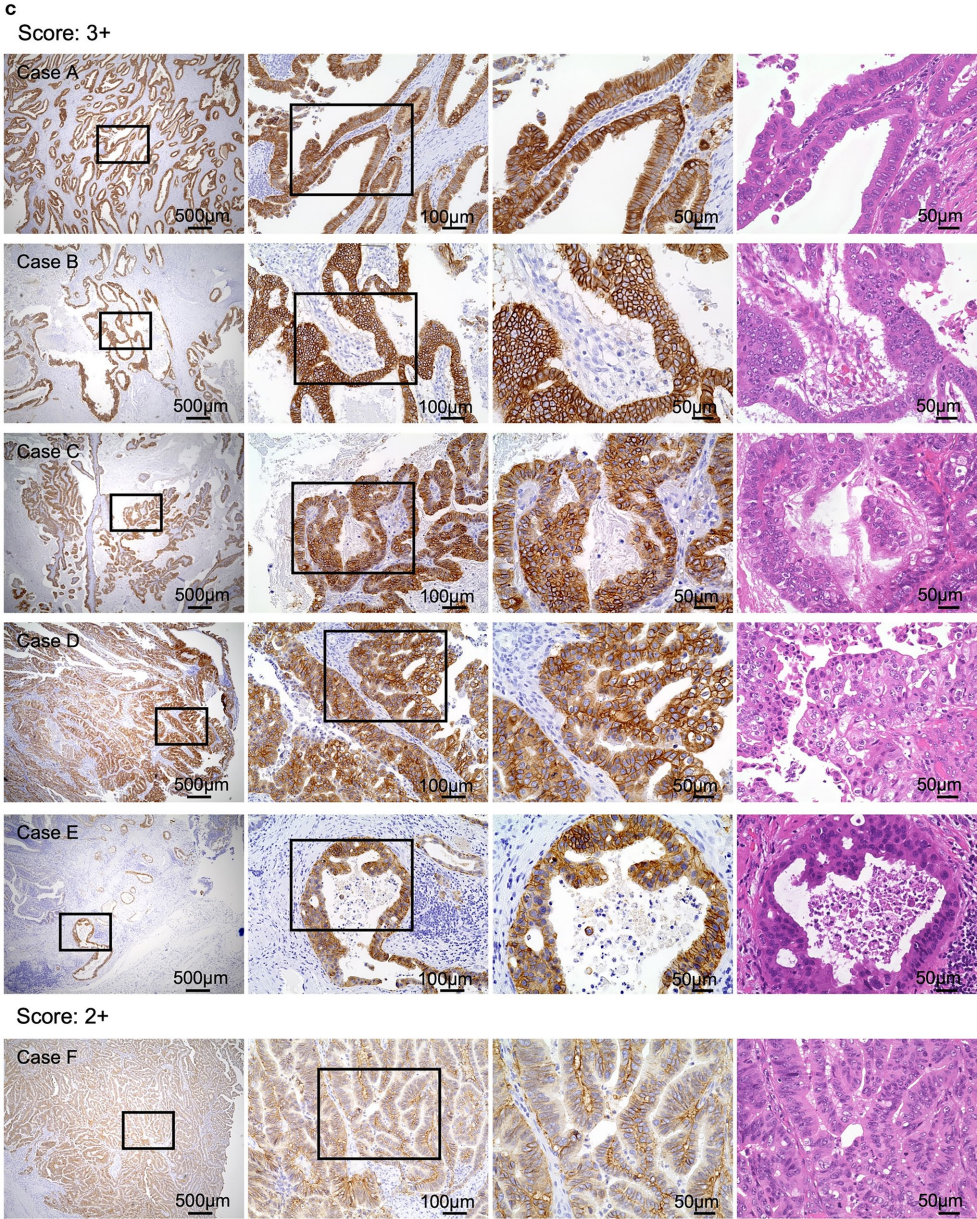

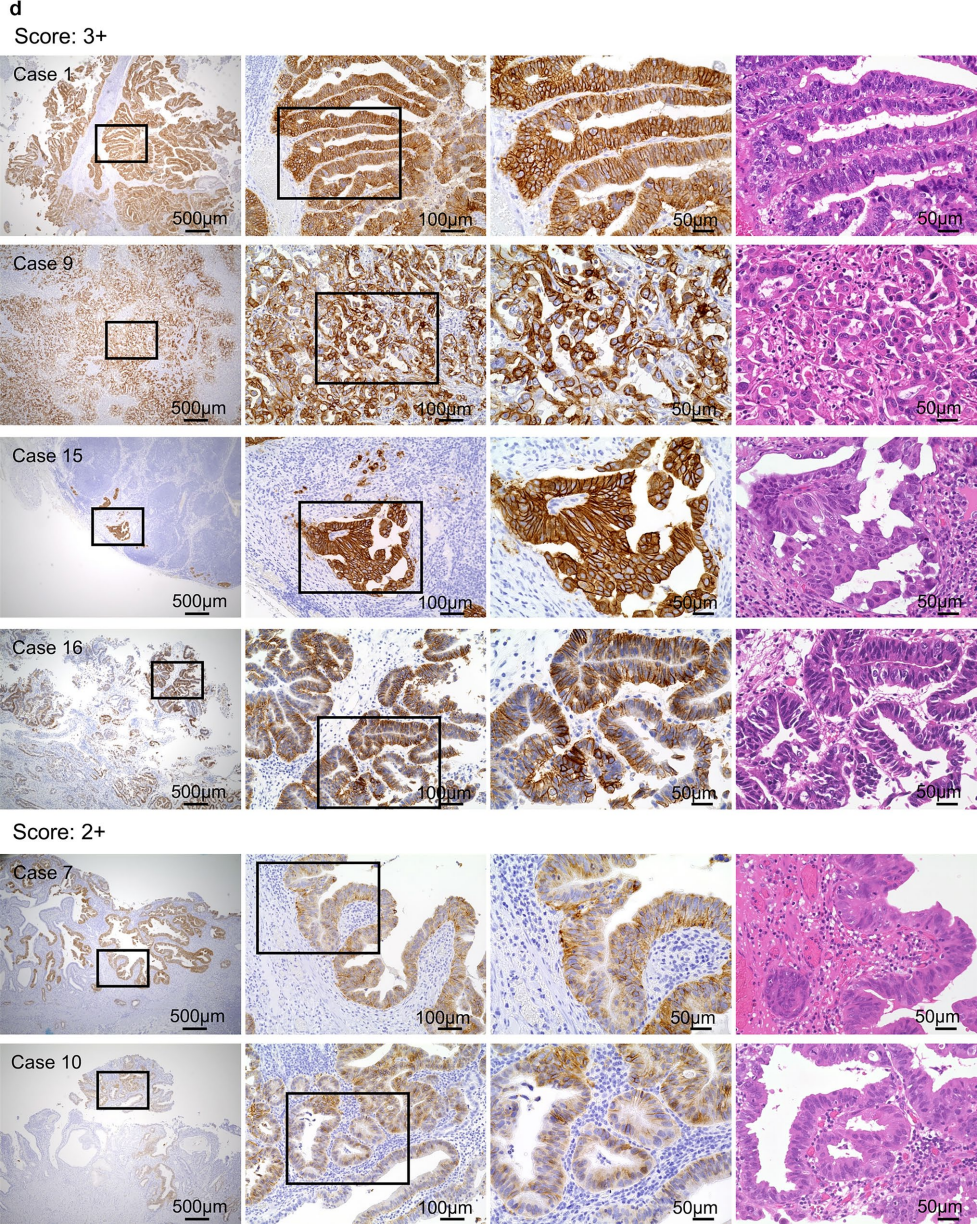
Comparison between patient groups. **a**, **b** A: Normal; B: PBM; C: GBC without PBM; D: GBC with PBM. No high positive rates were observed in the normal and PBM groups (Groups A and B). Among the 45 patients in the GBC without PBM group (Group C), seven (15.6%) had scores of ≥ 2 +. HercepTest scores ≥ 2 + were observed in eight (50%) patients in the GBC with PBM group (Group D), with detailed scoring proportions provided in (**b**). *P < 0.05, **P < 0.01, ****P < 0.0001**. c **Representative images of HER2 overexpression in Group C (GBC without PBM). **d** Representative images of HER2 overexpression in Group D (GBC with PBM). In each row, the four images are derived from the same case. The first three figures represent HercepTest staining of the same field under different magnifications, while the third and fourth figures correspond to HercepTest and H&E staining of the same field, respectively, at the same magnification. Boxes indicate enlarged regions. *GBC* Gallbladder cancer, *PBM* Pancreaticobiliary maljunction, *H&E* Hematoxylin and eosin, *HER2* Human epidermal growth factor receptor 2

As presented in Table S1, 15 (seven and eight cases from Groups C and D, respectively) of the 127 cases (11.8%) had HercepTest scores ≥ 2 +. Meanwhile, HercepTest results were negative in all patients of Groups A and B. The detailed statistical results are provided in Fig. 4a, b. The HER2 overexpression rate was significantly higher in Group D than in the other groups (*P* = 0.006, chi-squared test). Representative partially positive HercepTest and corresponding H&E staining images for Groups C and D are shown in Fig. 4c, d, respectively.

### FISH

**Fig. 5:**
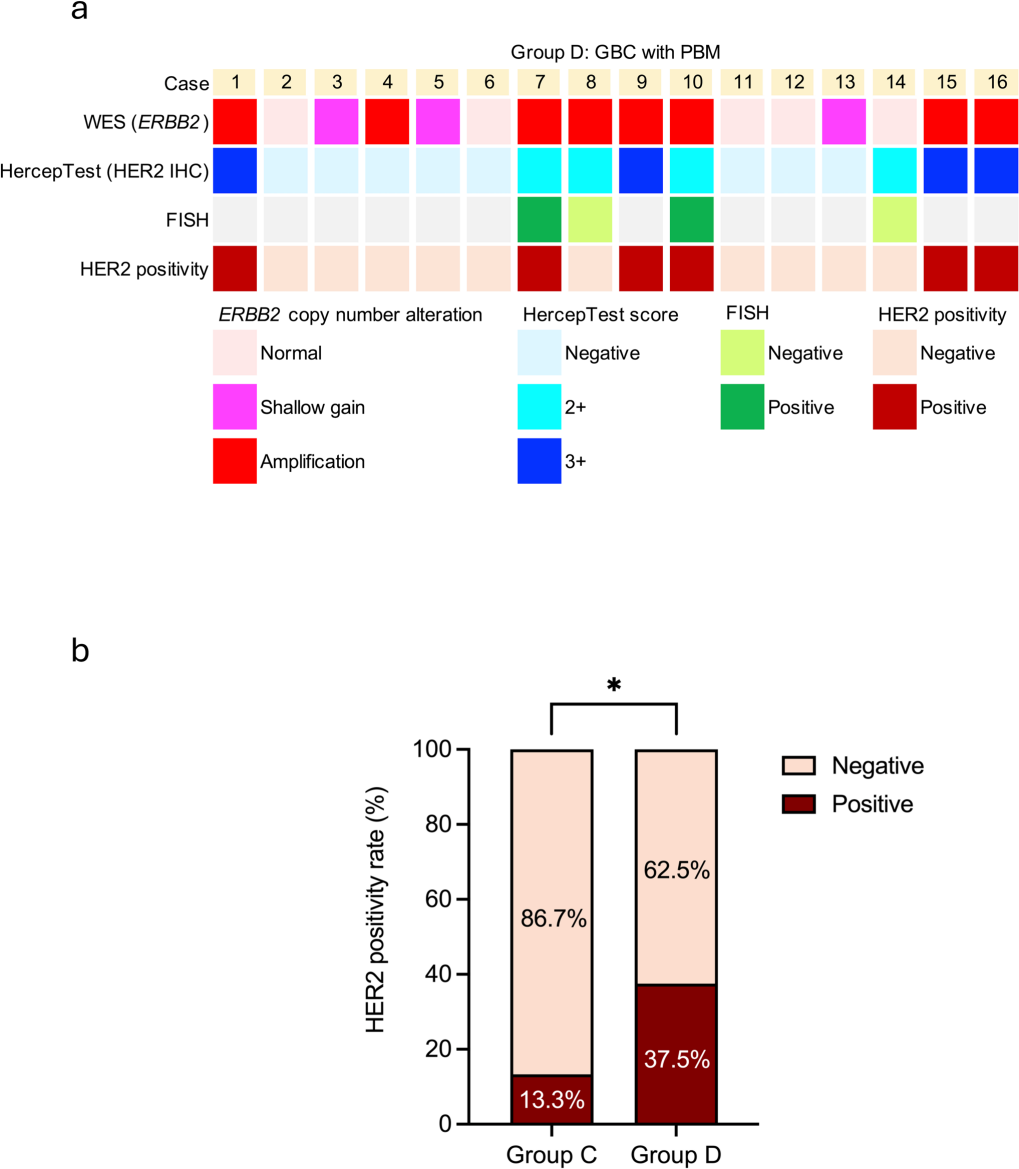
HER2 expression in tissues of GBC without PBM (Group C) and GBC with PBM (Group D). **a** Comparison of WES, HercepTest, and FISH results for 16 Group D patients. The copy number alteration rate for *ERBB2* was 68.8% (11/16), with an amplification rate of 50% (8/16). In addition, 50.0% of the patients had a HercepTest score of ≥ 2 + (8/16). Among the four cases subjected to FISH analysis, two were HER2-positive. **b** In the GBC group (Group C), a total of 45 cases were analyzed. The HER2 overexpression rate was 13.3% (6/45). Among the 16 cases of GBC with PBM (Group D), the HER2 overexpression rate was 37.5% (6/16). *P < 0.05. *GBC* Gallbladder cancer; *PBM* Pancreaticobiliary maljunction; *WES* Whole-exome sequencing; *FISH* Fluorescence in situ hybridization; *HER2* Human epidermal growth factor receptor 2; *ERBB2* Erb-b2 receptor tyrosine kinase 2

FISH analysis was conducted on two and four patients from Groups C and D, respectively (all had a HercepTest score of 2 +). In Group C, one patient was excluded because of inconclusive results, and another was confirmed positive (HER2:CEP17 ratio of ≥ 2:1). In Group D, two patients were confirmed positive (Figs. 5a and S2). HER2 expression was considered positive at an immunostaining score of 3 + or 2 + when gene amplification was confirmed through FISH. The proportions of positive cases in Groups C and D were 13.3% (6/45) and 37.5% (6/16), respectively (*P* = 0.037, chi-squared test) (Fig. 5b).

### Comparison of test results in GBC with PBM cases

The WES, HercepTest, and FISH results of the 16 patients in Group D are shown in Fig. 5a. WES showed *ERBB2* amplification in patients 1, 4, 7, 8, 9, 10, 15, and 16 and shallow gain in patients 3, 5, and 13. HercepTest scores of ≥ 2 + were observed in patients 1, 7, 8, 9, 10, 14, 15, and 16; the remaining patients were negative for HER2 expression. Among patients 7, 8, 10, and 14, positive FISH results were observed in patients 7 and 10, whereas the remaining patients (HercepTest scores of 2 +) tested HER2-negative. The combination of HercepTest and FISH results confirmed HER2 overexpression in six patients. Comparative analysis of the 16 patients revealed that, when grouped based on HER2 protein expression, *ERBB2* alterations were the only parameter reaching statistical significance, while other factors, including the presence or absence of bile duct dilatation, were not significant (Table S2).

### Enrichment analysis of RNA extracted from gallbladder mucosa in PBM

**Fig. 6:**
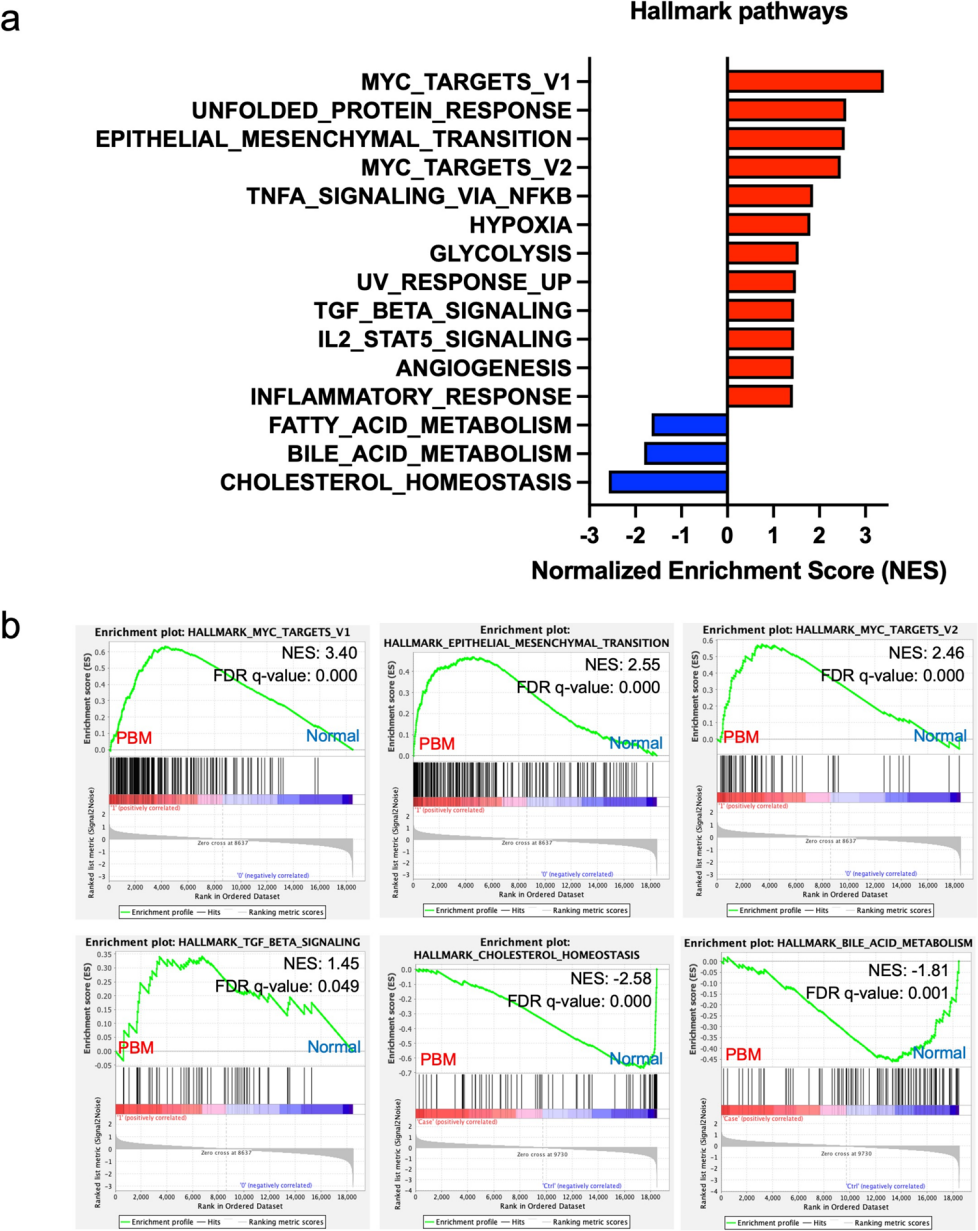
Normalized Enrichment Score (NES) analysis of hallmark pathways in PBM and normal controls. **a** Gene set enrichment analysis (GSEA) using hallmark gene sets from the Molecular Signatures Database (MSigDB). Red bars indicate the pathways enriched in the PBM, and blue bars represent those enriched in the normal (*n* = 3 for both groups). Statistically significant signatures were selected (false discovery rate (FDR) < 0.05). **b** GSEA showing enrichment of Myc target gene signature, epithelial–mesenchymal transition gene signature, DNA-repair gene signature, and TGF-β signaling gene signature in PBM and that of Cholesterol homeostasis and bile acid metabolism in normal controls. *PBM* Pancreaticobiliary maljunction;* TGF* Tumor growth factor

GSEA of hallmark gene sets following RNA-seq of gallbladder mucosal epithelium from three normal patients and three with PBM (Fig. 6a, b) showed upregulation of pathways related to MYC targets, epithelial–mesenchymal transition (EMT), hypoxia, tumor necrosis factor-α signaling via nuclear factor-kappa B (NF-κB), glycolysis, DNA repair, and tumor growth factor (TGF)-β signaling, among others. These pathways are associated with tumorigenesis, cell proliferation, inflammation, and metabolic reprogramming, all of which contribute to cancer development [38–42]. Conversely, the downregulated pathways, including bile acid metabolism and cholesterol homeostasis, suggest metabolic dysregulation, which can contribute to carcinogenesis [43]. Overall, the significant enrichment of oncogenic pathways in the gallbladder mucosa of patients with PBM suggests a higher probability of malignant transformation, supporting PBM as a high-risk condition for GBC development.

## Discussion

We performed a comprehensive molecular analysis of GBC with PBM to elucidate the potential relationships between tumor genotypic and clinical phenotypic features. Notably, we identified a high prevalence of *ERBB2* amplification (50.0%) and HER2 protein overexpression (37.5%) in this unique patient cohort. The relative concordance between *ERBB2* amplification and HER2 overexpression provides preliminary support for targeting these molecules in treating this patient population.

*ERBB2* alterations have been reported in 3.0–17.0% of GBC cases, most commonly associated with gene amplification [44]. The *ERBB2* amplification rate in GBC has been reported at 9.1% in Japan [45], while the globally weighted average amplification rate is 7.1% [46]. However, the molecular relationships between GBC and PBM remain largely unclear. Notably, the high *ERBB2* amplification rate in this GBC with PBM subgroup suggests a potential interplay between PBM and *ERBB2*-driven carcinogenesis.

PBMs are a congenital anomaly of the pancreaticobiliary junction, which can lead to the reflux of mixed pancreatic juice and bile into the gallbladder [11]. The carcinogenic mechanism likely involves the reflux of trypsin and transformed lysolecithin, which damage the epithelial cell membrane of the gallbladder [47]. Previous studies have demonstrated that prolonged exposure of the mucosal epithelium to bile acids and pancreatic enzymes can activate pro-oncogenic signaling pathways, such as NF-κB and TGF-β, thereby promoting EMT, cellular proliferation, and anti-apoptotic phenotypes [48, 49]. Our RNA-seq data showed significant upregulation of these pathways in the gallbladder mucosa of patients with PBMs. This chronic pro-inflammatory and regenerative microenvironment may create favorable conditions for HER2 pathway activation and *ERBB2* amplification.

Moreover, the upregulation of the hypoxia pathway suggests the presence of local tissue hypoxia. Previous studies have shown that hypoxic conditions can enhance HER2 transcriptional activity by upregulating hypoxia-inducible factor (HIF)−1α or HIF-2α, as reported in other gastrointestinal cancers [39] and breast cancer [50]. Glycolysis was also markedly upregulated, consistent with the mechanism by which HER2 promotes the Warburg effect [51]. In addition, DNA repair pathway activation indicated genomic instability, potentially contributing to HER2 amplification [40]. These findings support the hypothesis that microenvironmental alterations caused by PBM may promote *ERBB2* amplification and overexpression through epigenetic mechanisms.

HER2 overexpression is an important prognostic and predictive biomarker in many malignancies [52, 53]. Approximately 15–20% of breast cancers and 10–20% of gastric adenocarcinomas (especially those originating from the gastric cardia) exhibit HER2 overexpression [31, 52]. HER2 is reportedly overexpressed in 6.6–17.0% of GBC cases [21], consistent with the 19.7% (*n* = 12, 12/61) observed in this study. However, the markedly higher rate of HER2 overexpression (37.5%) observed in GBC with PBM cases supports the hypothesis that PBMs may promote a HER2-enriched oncogenic phenotype. Groups A and B, comprising patients without cancer, showed no HER2-positive expression, regardless of PBM status. The absence of HER2 staining in normal gallbladder epithelium is consistent with the findings of several previous studies on GBC and breast cancer [54, 55]. Overall, these earlier studies and the present study confirm that HER2 expression is low in normal gallbladder epithelial cells. Increased HER2 expression appears to emerge only when the epithelial cells progress to precancerous stages, including intestinal metaplasia, epithelial dysplasia, or carcinoma in situ [56]. Thus, the strong relationship with HER2 protein expression occurs only in the combined context of GBC and PBM.

WES and HercepTest analyses demonstrated a high degree of concordant results for *ERBB2* amplification and HER2 overexpression in Group D, corroborating the association between these alterations. HercepTest and FISH provide reliable methods for detecting HER2 overexpression or *ERBB2* amplification and are essential for identifying patients eligible for HER2-targeted therapies. Although a general concordance was observed between *ERBB2* amplification and HER2 overexpression, discrepancies among different detection methods were noted, with some cases of *ERBB2* amplification exhibiting negative HER2 protein expression (Fig. 5a). This may be because 1) WES is a more sensitive quantitative method, whereas HercepTest relies on qualitative scoring, and 2) WES captures the whole tumor genome, whereas HER2 expression may vary across tumor regions [57]. In addition, the HercepTest reportedly misses up to 22.7% of HER2-positive cases [34]. This HER2 detection method still has limitations in accurately identifying HER2 expression within cancerous tissues [58, 59]. Given the known limitations of HercepTest and FISH, integrating genomic analyses such as WES may enhance diagnostic accuracy and provide more precise guidance for therapeutic decision-making.

Trastuzumab (Herceptin), a monoclonal antibody, improves overall survival and reduces recurrence rates by targeting HER2 (*ERBB2*) as part of a standard chemotherapy regimen in patients with HER2-positive breast and gastric cancers [60]. Indeed, HER2-targeted therapies are invaluable for treating HER2-positive malignancies [61]. Although the U.S. Food and Drug Administration (FDA) has not yet approved HER2-targeted therapies specifically for GBC, early studies and case reports have demonstrated promising efficacy [62, 63]. Current clinical HER2-targeted therapy generally follows FDA recommendations, using HER2 protein overexpression as the primary criterion for determining eligibility for targeted therapy [35, 64]. However, emerging evidence suggests that some patients with low HER2 expression (defined as IHC 1 + or 2 + with negative ISH results) [58] may still derive clinical benefit from treatment with trastuzumab duocarmazine, with response rates reported in approximately 28–40% of cases [58, 59]. Considering that more than half of the Group D patients in this study exhibited HER2 overexpression or *ERBB2* amplification, routine assessment of HER2 status using HercepTest, FISH, and/or genomic analysis is recommended to identify candidates and approaches for HER2-targeted therapy.

This study had some limitations. First, the study cohort comprised a specific population treated at a single institution, reflecting only the characteristics of the northeastern Japanese population over the past 15 years, which may limit the generalizability of our findings. Second, the limited sample size, particularly in the GBC with PBM group, precluded robust intragroup statistical analyses and hindered comprehensive subgroup comparisons. Therefore, future studies involving multicenter, prospective cohorts with larger sample sizes, greater ethnic diversity, and standardized diagnostic algorithms are warranted to validate our findings.

In conclusion, this study highlights a potential molecular interplay between PBM and GBC, characterized by *ERBB2* amplification and HER2 overexpression. These findings support incorporating HER2-targeted strategies into the management of GBC with PBM and underscore the need for further studies on the underlying pathogenic mechanisms and therapeutic implications.

## Supplementary Information

Below is the link to the electronic supplementary material.

Supplementary file1 (DOCX 12160 KB)

## Abbreviations

GBC: Gallbladder cancer
PBM: Pancreaticobiliary maljunction
*ERBB2*: Erb-b2 receptor tyrosine kinase 2
*ERBB3*: Erb-b2 receptor tyrosine kinase 3
*ERBB4*: Erb-b2 receptor tyrosine kinase 4
*EGFR*: Epidermal growth factor receptor
HER2: Human epidermal growth factor receptor 2
*ELF3*: E74-like ETS transcription factor 3
ERCP: Endoscopic retrograde cholangiopancreatography
WES: Whole-exome sequencing
FFPE: Formalin-fixed paraffin-embedded
H&E: Hematoxylin and eosin
FISH: Fluorescence in situ hybridization
CEP17: Chromosome enumeration probe 17
ASCO/CAP: American society of clinical oncology/college of American pathologists
RNA-seq: RNA sequencing
GSEA: Gene set enrichment analysis
MSigDB: Molecular signatures database
DEG: Differentially expressed gene
GO: Gene ontology
*TP53*: Tumor protein p53
*KRAS*: Kirsten ras
EMT: Epithelial–mesenchymal transition
NF-κB: Nuclear factor-kappa B
TGF: Tumor growth factor
