# Correction: Molecular insights into HER2/*ERBB2* amplification and carcinogenesis in gallbladder cancer associated with pancreaticobiliary maljunction

**DOI:** 10.1007/s00535-025-02329-6

**Published:** 2025-11-26

**Authors:** Ming Zhu, Daisuke Douchi, Keigo Murakami, Taito Itoh, Shusuke Migita, Naoki Rikiyama, Shuichiro Hayashi, Takashi Kokumai, Hideaki Sato, Shingo Yoshimachi, Akiko Kusaka, Mitsuhiro Shimura, Shun Nakayama, Shuichi Aoki, Masahiro Iseki, Takayuki Miura, Shimpei Maeda, Masaharu Ishida, Hideo Ohtsuka, Masamichi Mizuma, Kei Nakagawa, Atsushi Masamune, Toru Furukawa, Michiaki Unno

**Affiliations:** 1 Department of Surgery, Tohoku University Graduate School of Medicine, 1-1 Seiryo-Machi, Aoba-Ku, Sendai, Miyagi Japan; 2 Department of Investigative Pathology, Tohoku University Graduate School of Medicine, 1-1 Seiryo-Machi, Aoba-Ku, Sendai, Miyagi Japan; 3 Laboratory of Molecular Diagnostics and Therapeutics, Center for Intractable Diseases and ImmunoGenomics, National Institutes of Biomedical Innovation, Health and Nutrition, 7-6-8 Saito-Asagi, Ibaraki, Osaka Japan; 4 Division of Gastroenterological and Hepato-Biliary-Pancreatic Surgery, Department of Surgery, Tohoku Medical and Pharmaceutical University, 1-15-1 Fukumuro, Miyagino-Ku, Sendai, Miyagi Japan; 5 Division of Gastroenterology, Tohoku University Graduate School of Medicine, 1-1 Seiryo-Machi, Aoba-Ku, Sendai, Miyagi Japan

**Correction: J Gastroenterol** 10.1007/s00535-025-02303-2

The Data Transparency statement was missing and should have read as given below.

**Data Transparency Statement** The RNA-seq data generated in this study have been deposited in the NCBI Sequence Read Archive (SRA) under the BioProject accession number PRJNA1320976 and are publicly accessible. Due to ethical restrictions related to human genomic data, the raw WES data are not publicly available. These DNA sequencing datasets can be obtained from the corresponding author upon reasonable request, in accordance with the institutional ethics approval.

