# Supplementary material for: Molecular insights into HER2/ERBB2 amplification and carcinogenesis in gallbladder cancer associated with pancreaticobiliary maljunction
Source: J Gastroenterol. 2025 Oct 4;61(2):222–38. doi: 10.1007/s00535-025-02303-2 (PMC12924843; doi:10.1007/s00535-025-02303-2)
Supplement: Supplementary file 1 — Supplementary file1 (DOCX 12160 KB) [file 535_2025_2303_MOESM1_ESM.docx]

**Supplementary Figures and Tables:**

Molecular Insights into HER2/*ERBB2* Amplification and Carcinogenesis in Gallbladder Cancer Associated with Pancreaticobiliary Maljunction

Ming Zhu MD*; Daisuke Douchi MD, PhD*; Keigo Murakami MD, PhD^†^; Taito Itoh MD, PhD^†,‡^; Shusuke Migita MD*; Naoki Rikiyama MD*; Shuichiro Hayashi MD*; Takashi Kokumai MD, PhD*; Hideaki Sato MD, PhD*; Shingo Yoshimachi MD, PhD*; Akiko Kusaka MD, PhD*; Mitsuhiro Shimura MD, PhD^*^; Shun Nakayama MD, PhD*; Shuichi Aoki MD, PhD*; Masahiro Iseki MD, PhD*; Takayuki Miura MD, PhD*; Shimpei Maeda MD, PhD*; Masaharu Ishida MD, PhD*; Hideo Ohtsuka MD, PhD*; Masamichi Mizuma MD, PhD*; Kei Nakagawa MD, PhD^§^; Atsushi Masamune MD, PhD^//^; Toru Furukawa MD, PhD^†^; Michiaki Unno MD, PhD*

* Department of Surgery, Tohoku University Graduate School of Medicine, 1-1 Seiryo-machi, Aoba-ku, Sendai, Miyagi, Japan

^†^ Department of Investigative Pathology, Tohoku University Graduate School of Medicine, 1-1 Seiryo-machi, Aoba-ku, Sendai, Miyagi, Japan

^‡^ Laboratory of Molecular Diagnostics and Therapeutics, Center for Intractable Diseases and ImmunoGenomics, National Institutes of Biomedical Innovation, Health and Nutrition, 7-6-8 Saito-Asagi, Ibaraki, Osaka, Japan

^§^ Division of Gastroenterological and Hepato-Biliary-Pancreatic Surgery, Department of Surgery, Tohoku Medical and Pharmaceutical University, 1-15-1 Fukumuro, Miyagino-ku, Sendai, Miyagi, Japan

^//^ Division of Gastroenterology, Tohoku University Graduate School of Medicine, 1-1 Seiryo-machi, Aoba-ku, Sendai, Miyagi, Japan

^*^**Corresponding Author:** Daisuke Douchi

Department of Surgery, Tohoku University Graduate School of Medicine

1‑1 Seiryo‑machi, Aoba‑ku, Sendai, Miyagi 980‑8574, Japan

Tel: +81-22-717-7201

FAX: +81-22-717-7209

E-mail: [daisuke.douchi.e6@tohoku.ac.jp](mailto:daisuke.douchi.e6@tohoku.ac.jp)

ORCiD: 0000-0003-4582-6589

**Supplementary Table S1:**

**HercepTest scoring results of four groups**

|  | A  Normal | B  PBM | C  GBC without PBM | D  GBC with PBM |
| --- | --- | --- | --- | --- |
| Negative (0 or 1+) | 33 | 33 | 38 | 8 |
| 2+ | 0 | 0 | 2 | 4 |
| 3+ | 0 | 0 | 5 | 4 |
| Total | 33 | 33 | 45 | 16 |

Normal means Control group (chronic cholecystis); PBM: pancreaticobiliary maljunction; GBC: Gallbladder Cancer; The distribution of patients with different HercepTest scores across the four groups.

**Supplementary Table S2:**

**Clinicopathological & molecular characteristics of GBC with PBM patients**

This table summarizes detailed clinicopathological data and molecular test results for the 16 patients classified as Group D in the present study. Variables include patient demographics, histological subtypes, TNM staging, and the results of HER2 immunohistochemistry (HercepTest), fluorescence in situ hybridization (FISH), and *ERBB2* copy number alterations obtained by whole-exome sequencing (WES), ^a^: Fisher’s exact test, ^b^: Kruskal–Wallis H test.

**Supplementary Figure S1:**


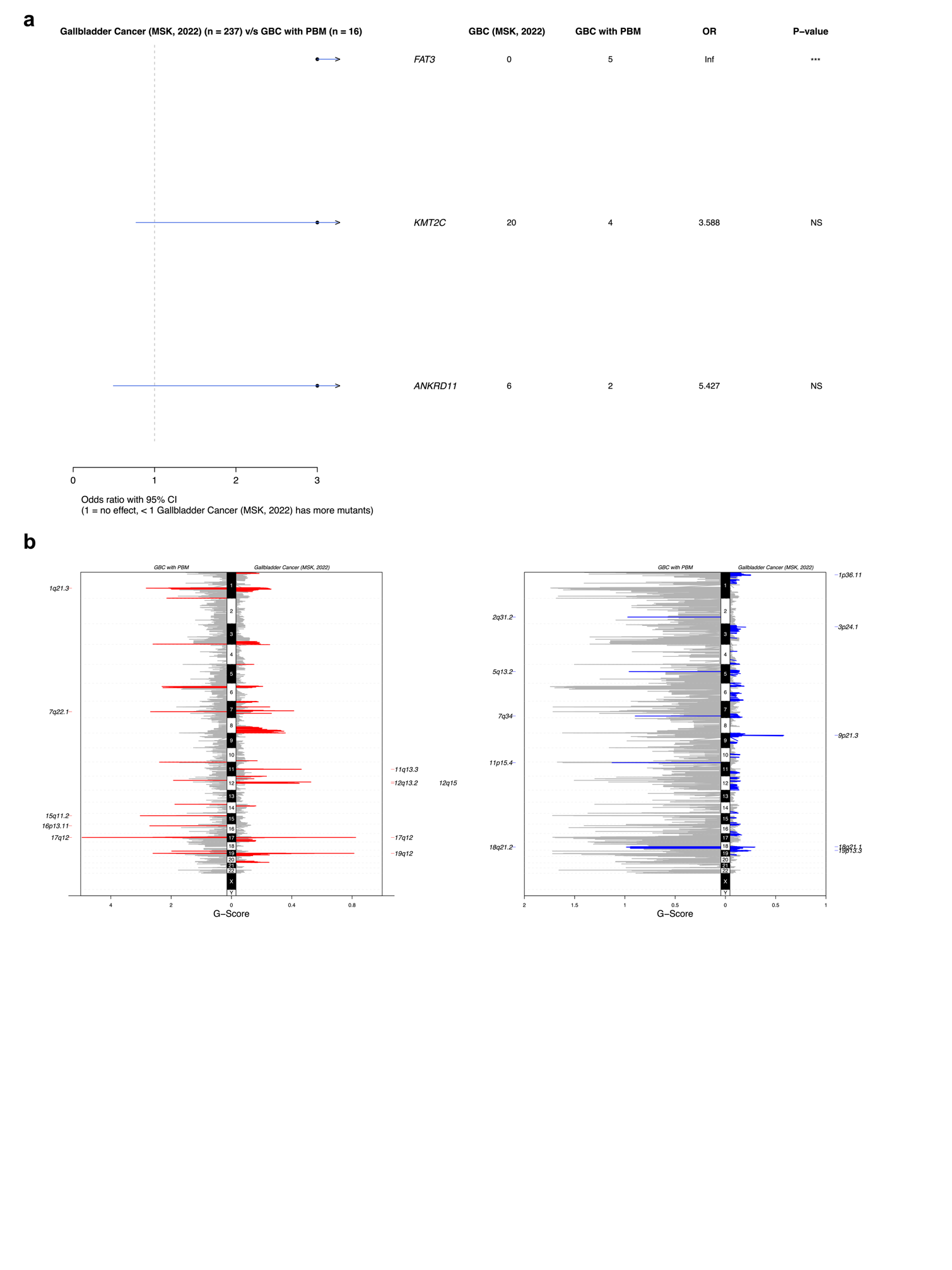


**Figure S1: Comparison of somatic mutation enrichment and focal copynumber alterations in GBC with PBM versus a reference MSK cohort.**

(a) Forest plot of gene level mutation frequencies. For each gene, the mutation count in the MSK gallbladder cancer cohort (n = 237) and the GBC with PBM cohort (n = 16) is shown alongside the odds ratio (OR) with 95% confidence interval and associated P-value. Confidence intervals that extend beyond the x-axis are indicated by arrows; “Inf” denotes an infinite OR arising from zero events in the reference cohort. “NS” indicates a non significant P-value and “***” indicates P < 0.001.

(b) Genome-wide GISTIC2.0 G-score plots of focal somatic copy-number alterations. The left panel displays G-scores for recurrent amplifications in GBC with PBM cohort (left side) compared with the MSK cohort (right side); the right panel shows recurrent deletions in GBC with PBM (left side) versus MSK (right side). Prominent peaks are annotated by cytogenetic band.

All panels were generated using the maftools package.

GBC: gallbladder cancer; PBM: pancreaticobiliary maljunction; MSK, Memorial Sloan Kettering

**Supplementary Figure S2:
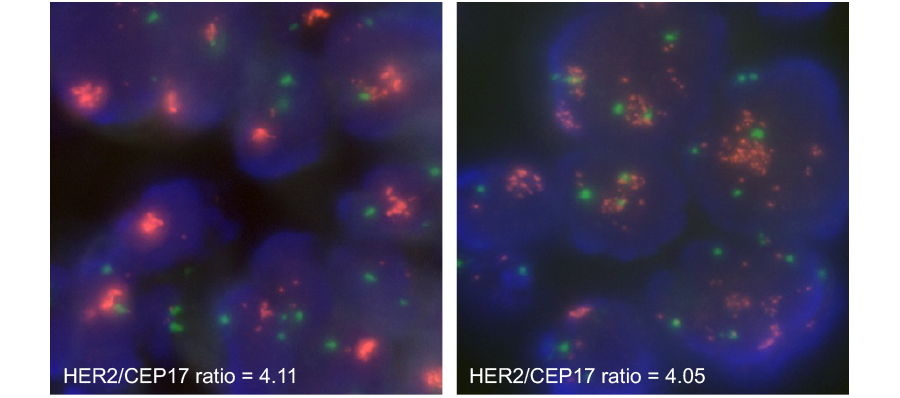
**

**Figure S2: Representative HER2 fluorescence in situ hybridization (FISH) assay images of tissues in the GBC with PBM group.** Red signals represent the HER2 gene (*ERBB2*) locus. Green signals represent the centromere of chromosome 17 (CEP17), which serves as a reference control. Blue background represents the DAPI-stained nuclei.

GBC: gallbladder cancer; PBM: pancreaticobiliary maljunction; HER2: human epidermal growth factor receptor 2; *ERBB2*: erb-b2 receptor tyrosine kinase 2; DAPI, 4′,6-diamidino-2-phenylindole
